# Parental behaviour and family proximity as key to gosling survival in Greylag Geese (*Anser anser*)

**DOI:** 10.1007/s10336-019-01638-x

**Published:** 2019-02-20

**Authors:** Georgine Szipl, Alina Loth, Claudia A. F. Wascher, Josef Hemetsberger, Kurt Kotrschal, Didone Frigerio

**Affiliations:** 1 Core Facility KLF for Behaviour and Cognition, University of Vienna, Fischerau 11, 4645 Gruenau im Almtal, Austria; 2 Department of Behavioural Biology, University of Vienna, Althanstrasse 14, 1090 Vienna, Austria; 3 University of St Andrews, North Haugh, St Andrews, KY16 9ST Scotland UK; 4 School of Life Sciences, Anglia Ruskin University, East Road, Cambridge, CB1 1PT UK

**Keywords:** Parental behaviour, Gosling survival, Greylag Goose, *Anser anser*

## Abstract

Reproductive success in monogamous species is generally affected by both behavioural and hormonal fine-tuning between pair partners. Vigilance, defence and brooding of offspring are among the main parental investments, and often the sexes adopt different roles. In the present study, we investigate how sex differences in parental behaviour and family proximity in the socially monogamous Greylag Goose (*Anser anser*) affect gosling survival. During the reproductive season in spring 2013, we recorded the behaviour of 18 pairs with offspring and gosling survival in a semi-tame, long-term monitored, and individually marked flock of Greylag Geese in Grünau, Austria. We found that behavioural role differentiation between the parents varied with developmental phase, and thus with gosling age. Especially during the first 10 days after hatching, females were foraging more frequently than males, which were more vigilant and aggressive towards other flock members. Such differences between the sexes levelled out 20 to 30 days after hatching. In general, females stayed in closer proximity to their offspring than males. Gosling survival was high when the parents were relatively aggressive and emphasized vigilance rather than foraging behaviour. Hence, we show a direct link between pair partners’ quality of parental investment and gosling survival.

**Electronic supplementary material:**

The online version of this article (10.1007/s10336-019-01638-x) contains supplementary material, which is available to authorized users.

## Zusammenfassung

**Elterliches Verhalten und familiäre Nähe als Schlüssel zum Überleben in Graugänsen (** ***Anser anser*** **)**

Der Reproduktionserfolg bei monogamen Arten wird im Allgemeinen von der Feinabstimmung der Paarpartner im Verhalten sowie auf hormoneller Ebene beeinflusst. Wachsamkeit, Verteidigung und das Bebrüten des Nachwuchses gehören zu den wichtigsten Investitionen der Eltern, und oft nehmen die Geschlechter unterschiedliche Rollen ein. In der vorliegenden Studie untersuchten wir, wie sich geschlechtsspezifische Unterschiede im elterlichen Verhalten und die Nähe der Familienmitglieder zueinander in der sozial monogamen Graugans (*Anser anser*) auf das Überleben der Jungvögel auswirken. Während der Fortpflanzungssaison im Frühjahr 2013 haben wir das Verhalten von 18 Paaren mit Nachkommen und das Überleben der Jungvögel in einer halbzahmen, langzeitüberwachten und individuell markierten Schar von Graugänsen in Grünau/Österreich aufgezeichnet. Wir stellten fest, dass die Differenzierung der Verhaltensrolle zwischen den Eltern mit der Entwicklungsphase der Jungvögel und somit mit deren Alter variierte. Insbesondere während der ersten zehn Tage nach dem Schlüpfen waren Weibchen häufiger auf Nahrungssuche als Männchen, die hingegen wachsamer und aggressiver gegenüber anderen Mitgliedern der Schar waren. Diese Unterschiede zwischen den Geschlechtern glichen sich zwanzig bis dreißig Tage nach dem Schlupf aus. Im Allgemeinen blieben die Weibchen näher bei ihren Nachkommen als die Männchen. Das Überleben der Jungvögel war hoch, wenn die Eltern relativ aggressiv waren und mehr Wert auf Wachsamkeit als auf Nahrungssuche legten. Daher zeigen wir eine direkte Verbindung zwischen der Qualität der elterlichen Investitionen der Paarpartner und dem Überleben der Jungvögel.

## Introduction

In waterfowl, the number of offspring at hatching and fledging are among the most often used measures of fitness (Lack 1966; Trivers 1972; Lamprecht 1986; Cooke and Rockwell 1988; Williams et al. 1994; Cooke et al. 1995). In long-term monogamous biparental birds, reproductive success is affected by factors such as social status and/or breeding experience (Lamprecht 1986; Black and Owen 1989; Lamprecht 1990; Forslund 1993; Nilsson and Persson 1994; Black et al. 2007) and pair bond duration (Fowler 1995; Black et al. 1996; van de Pol et al. 2006; Naves et al. 2007), as well as social and hormonal compatibility between partners (Choudhury et al. 1996; Spoon et al. 2006; Weiss et al. 2010; Hirschenhauser 2012). Clearly, the coordinated interactions of pair partners, including the quality and timing of specific parental behaviour, affect gosling survival and, therefore, reproductive success (Lamprecht 1990; Williams et al. 1994).

Furthermore, in precocial species, parents do not feed their offspring but rather lead them to appropriate feeding areas, thereby reducing competition over food with conspecifics, maintaining family cohesion and avoiding predators (Lazarus and Inglis 1978; Schindler and Lamprecht 1987). The main components of parental care in precocial birds are vigilance, defensive behaviour and brooding of the goslings (Black and Owen 1989; Williams et al. 1994). In Barnacle Geese (*Branta leucopsis*), for instance, both parental males and parental females spend much more time being vigilant than males and females without goslings (Black and Owen 1989; Forslund 1993; Black et al. 2007). Moreover, parents adjust their activity budgets to the number of goslings, where parents with larger families spend less time foraging than parents with small families [e.g. Bar-headed Goose, *Anser indicus* (Schindler and Lamprecht 1987); Barnacle Goose (Forslund 1993; Siriwardena and Black 1998; Loonen et al. 1999); Lesser Snow Goose, *Chen caerulescens caerulescens* (Cooke and Rockwell 1988; Williams et al. 1994)].

In Greylag Geese, the coordination in specific behavioural combinations may influence gosling survival, e.g. the female foraging and the male being vigilant, or both pair partners resting simultaneously (Nedelcu and Hirschenhauser 2013). However, few studies investigate behavioural differences between the sexes in waterfowl during the parental phase (Sedinger and Raveling 1990; Fraser et al. 2002). Alongside behavioural coordination, spatial proximity between individuals may be an important factor contributing to successful reproduction in waterfowl. Besides ecological factors such as predation avoidance (Davies et al. 2012), there are well-documented social aspects influencing spatial proximity within a group, and it is generally assumed that dyadic distances usually reflect social cohesion in social mammals (Hinde and Atkinson 1970; Hinde 1983) and in birds (Frigerio et al. 2001; Black and Owen 1995; Black 1998). Tolerance between individuals in close spatial proximity is an important indicator of a pair bond in monogamous waterfowl, and particularly in geese (Lorenz 1988), and is associated with several functional benefits, including increased success in agonistic encounters (Scott 1980) and buffering of the physiological stress response (Wascher et al. 2012). Yet little is known about the spatial proximity between parents and their goslings and how it influences gosling survival.

During the reproductive season in spring 2013, we investigated the behaviour of parental geese and proximity within the families and linked it to gosling survival in the long-term monitored and individually marked flock of Greylag Geese in Grünau im Almtal (Austria). We hypothesized behavioural differences between male and female parents and an additional effect of the number and the developmental phase (i.e. the age) of the goslings. In general, we expected males to be more aggressive than females. Furthermore, according to previous findings in the Lesser Snow Goose (Williams et al. 1994) and Cackling Canada Goose (Sedinger and Raveling 1990), we expected females to forage more in order to regain weight lost during incubation, while males would be more vigilant. With respect to the number of goslings, we expected to find more vigilance and more aggressive behaviour when the number of goslings was high, while foraging would be reduced with higher numbers of offspring, as it constrains vigilance [e.g. Bar-headed Goose (Schindler and Lamprecht 1987); Barnacle Goose (Forslund 1993; Siriwardena and Black 1998; Loonen et al. 1999); Lesser Snow Goose (Williams et al. 1994; Black et al. 2007)]. Behavioural differences between sexes were expected to be highest during the early rearing phase and to gradually diminish as goslings grew older and relied less on parental investment. Regarding family proximity, females were expected to stay closer to their goslings than males. This expectation is based on previous studies, which showed a major role for the female in maintaining family cohesion within the flock (Scheiber and Weiss 2013). We expected to find a relationship between parental behaviour and gosling survival, i.e. more vigilant and aggressive parents are expected to raise more offspring.

## Methods

### Study area and focal animals

The Konrad Lorenz Research Station (KLF) is located 550 m above sea level in a valley in the northern part of the Austrian Alps (47°51N 13°57E). The studied non-migratory flock of Greylag Geese was introduced into the Upper Austrian valley of the river Alm by Konrad Lorenz and co-workers in 1973 (Lorenz 1988). The geese are unrestrained and generally spend the days on meadows and ponds close to the research station, where they are provided with supplemental food twice a day year-round. At night, the birds roost on ponds approximately 2 km to the south at the Cumberland Game Park. The flock is subject to natural selection: natural predators (mainly red foxes and golden eagles) may account for a loss of up to 10% of the flock per year (Hemetsberger 2001, 2002). All individuals are marked with coloured leg rings and are habituated to the close presence of humans. They show neither increased excreted immunoreactive corticosterone metabolites in the faeces nor modifications of the heart rate when approached by familiar humans (Scheiber et al. 2005; Wascher et al. 2011). Social behaviour and individual life history have been monitored since 1973. At the time of data collection, the flock consisted of 159 birds.

In the present study, a total of 120 Greylag Geese, i.e. 36 individually marked geese and their unmarked and unsexed offspring (*N*_adult__females_ = 18, *N*_adult__males_ = 18, *N*_goslings_ = 84) were observed from 14 April until 11 June 2013. The number of goslings per family ranged from 1 to 11 (mean ± SE = 4.83 ± 0.54; *N*_families_ = 18) at the beginning of the study and from 1 to 5 goslings (mean ± SE = 2.31 ± 0.38; *N*_families_ = 13) at the end of the study. The age of the observed adult geese ranged between 4 and 20 years for the males (mean ± SE = 10.78 ± 1.095) and between 4 and 18 years for the females (mean ± SE = 9.5 ± 1.076). Twenty-eight out of 36 parental geese (i.e. 77.7%) already had breeding experience (i.e. at least one-time hatched eggs), and 18 of them (i.e. 50%) had already fledged goslings at least once.

### Behavioural observations

Scan samples were performed every 30 s for 10 min (Martin and Bateson 1986) on the behaviour of both pair partners (i.e. the parents) simultaneously. According to the information collected during previous studies, the observation period was divided into three meaningful phases depending on the age of the goslings: phase 1 ranged from hatching to 5 days old; phase 2 from 10 to 15 days old and phase 3 from 25 to 30 days old (Hemetsberger 2002). A pair which lost all goslings was not observed further. Overall, a sum of 276 scans were conducted per individual (mean number of scans per phase per individual ± SE = 5.87 ± 0.15; for details see Supplemental Materials).

The following behaviours were recorded: (1) status signalling display, described as “beak up” (Lazarus and Inglis 1978; Lorenz 1988; Waldenberger and Kotrschal 1993); (2) vigilance behaviour: “head low”, “head up”, and “extreme head up” (Lazarus 1978; Lazarus and Inglis 1978; Lorenz 1988); (3) agonistic interactions, such as threats, attacks or fights (Raveling 1970; Lorenz 1988), distinguishing whether the focal goose was the initiator or receiver of an interaction; and (4) foraging behaviour. Behavioural observations were conducted by AL. Inter-observer reliability was established by coding behaviours from videos with GS, and reliability was excellent (ICC between 0.836 and 0.968 (Loth et al. 2017).

### Proximity data

In addition to behavioural observations, proximity measures were collected from the focal goose families via random and instantaneous spot checks (Martin and Bateson 1986). In total, 756 proximity measures were collected from the 18 focal families (mean ± SE = 42.0 ± 3.03; for details see Supplemental Materials). The observation period was divided into the same three phases as described above for the behavioural observations. Likewise, a pair was no longer observed when it had lost all its goslings. Active and inactive periods were considered separately. A goose family was defined as inactive when neither of the parents was vigilant, i.e. both parents were either resting or sleeping. Any other situation was considered as active. The minimum distances (d) between the mother and the father and the gosling closest to them, respectively, were estimated according to the following categories: *d* < 0.2 m; 0.2 m ≤ *d* < 0.5 m; 0.5 m ≤ *d* < 1 m; 1 m ≤ *d* < 1.5 m; 1.5 m ≤ *d* < 2 m; 2 m ≤ *d* < 2.5 m; ≥ 2.5 m. As brooding of the goslings occurs only at the beginning of the rearing period (Lorenz 1988), this behaviour was excluded from the analysis. In order to obtain independent data, an elapsed time of at least 15 min was assumed between two consecutive observations of the same family. Since the use of a laser distance measuring device would disturb the flock and therefore affect data collection (own experience), distances were estimated by AL. The estimation of the distances was trained with goose-sized objects (buckets and a tape, estimation error 15%) in order to gain reliable measurements of the distances.

### Statistical analysis

A principal component analysis (PCA) was conducted to reduce the number of behavioural variables using the packages GPA rotation (Bernaards and Jennrich 2005) and psych (Revelle 2017) in R (R Core Team 2018). Three principal components (PCs) with eigenvalues above 1.0 were extracted and varimax-rotated. PC1 explained 21% of the variance and comprised the frequencies of head low and head up, and was thus termed the “general vigilance” component. PC2 included the frequencies of beak up, threats and attacks (hereafter “agonistic interactions” component), and also explained 21% of the variance. The frequencies of foraging and extreme head up loaded on PC 3 and explained 17% of the variance. While foraging had a strong positive loading, extreme head up showed a negative loading (Table 1). This component illustrates the main trade-off/constraint between being vigilant (i.e. having the head up) and feeding with the head on the ground, and was termed the “foraging/head up” component. The individual regression scores for all three PCs were extracted for further analysis.

**Table 1:** Standardized loadings derived from the PCA (KMO = 0.61)

Behavioural variables	Principal components		
	PC1	PC2	PC3
Head low	**0.76**	− 0.08	0.02
Head up	**0.67**	0	− 0.01
Beak up	0.28	**0.71**	0.08
Threats	− 0.2	**0.77**	0.06
Attacks	− 0.07	**0.55**	− 0.09
Foraging	− 0.41	− 0.17	**0.76**
Extreme head up	− 0.39	− 0.16	− **0.77**
% of variance explained	21	21	17

Loadings higher than 0.5 are highlighted in bold

*KMO* Kaiser–Meyer–Olkin

To investigate the effects of phase, sex, and the number of goslings on the PCs, linear mixed-effects models (LMMs) with a normal distribution and an identity link were calculated for each PC separately using the package lme4 (Bates et al. 2015). A random effect was added that nested individuals within each family to account for repeated sampling. Phase, sex and the number of goslings, as well as the two-way interaction between phase and sex, were entered in the model as fixed effects. Models were ranked based on the difference in the corrected Akaike information criterion (ΔAICc), which was calculated by subtracting the lowest AICc from the respective AICc using the package AICcmodavg (Mazerolle 2017). In addition, relative likelihood [exp (−0.5/ΔAICc)] and Akaike weights (relative likelihood/sum of all relative likelihoods) were computed (Burnham et al. 2011). The models with the highest support were selected based on ΔAICc values ≦ 2 (see Table S1 in the Supplemental Materials). When several models had high support, model averaging was conducted with the package MuMIn (Bartoń 2016). Diagnostic plots were inspected to ensure that model assumptions were met and residuals were normally distributed.

To examine the effects of phase, sex and the number of goslings on the minimum distance between the parents and their goslings, an ordered probit regression, referred to as the cumulative link mixed model (CLMM) in the ordinal package (Christensen 2005) in R (R Core Team 2018), was used. Separate regressions were conducted for active and inactive periods. Individuals nested within each family were entered in the models as random effect. Phase, sex and the number of goslings, as well as the two-way interaction between phase and sex, were used as fixed effects. Models were ranked and—if necessary—averaged as described above. Model selection is shown in Table S2 in the Supplement.

The effects of parental behaviour on gosling survival were analysed with a weighted binomial logistic regression model in R (R Core Team 2018). A vector created from the number of goslings alive and the number of goslings lost during rearing was used as response variable. The individuals nested within each family were used as random effect. An additional random effect that included a unique value for each observation was added to account for overdispersion. The three PCs were entered as fixed effects. The full model explained variation in the data significantly better than the null model (likelihood ratio test: *γ*^2^ = 14.275, *df* = 3, *p* = 0.0026), and thus only the full model is presented the in “Results” section.

## Results

**Fig. 1:**
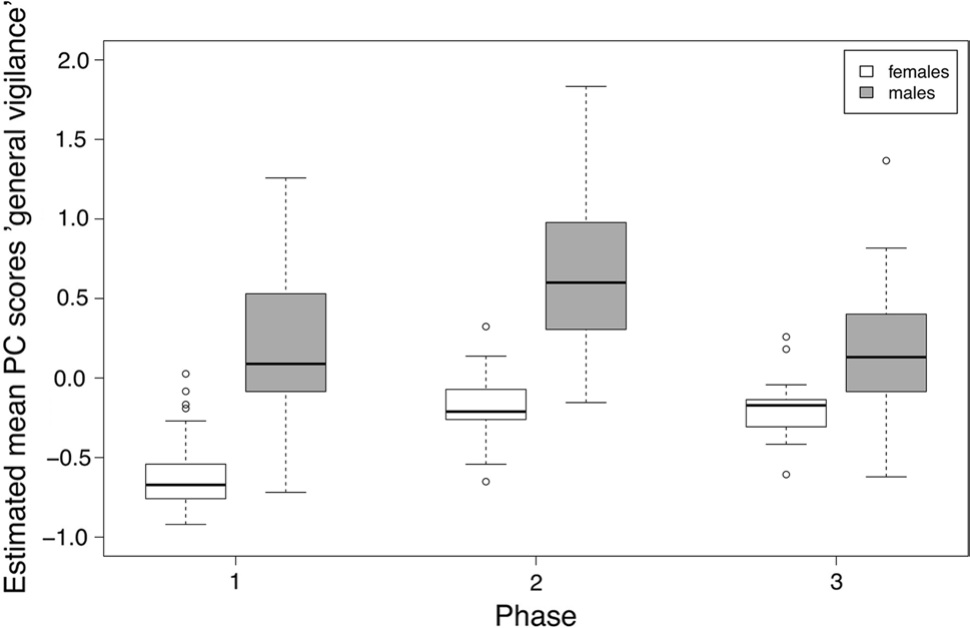
Estimated mean values for PC scores of general vigilance in male and female parental geese during the three phases of gosling rearing. General vigilance increased in phase 2 and decreased again in phase 3 in males, while females stayed equally alert in phases 2 and 3, although to a lesser extent than males

**Fig. 2:**
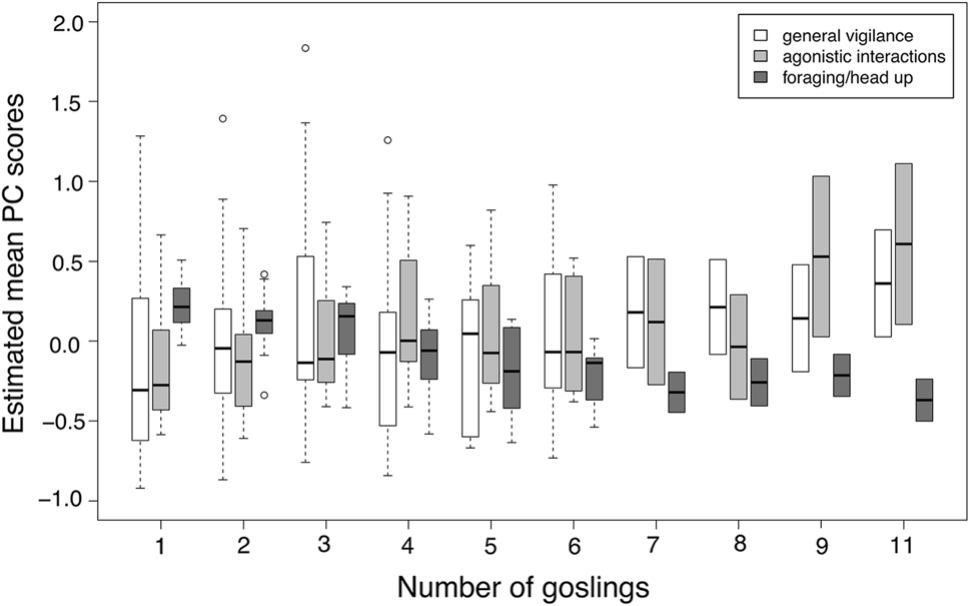
Estimated mean PC scores for the three PCs “general vigilance” (white boxes), “agonistic interactions” (light grey boxes) and “foraging/head up” (dark grey boxes) with respect to the number of goslings. While “general vigilance” and “agonistic interactions” increased with the number of goslings, scores of the “foraging/head up” decreased

**Fig. 3:**
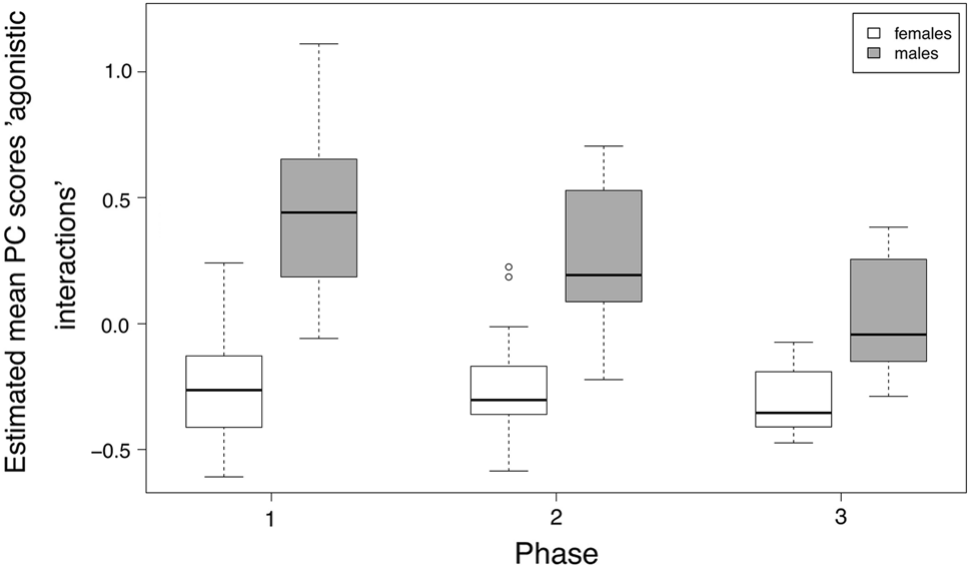
Estimated mean scores for agonistic interactions in female (white fills) and male (grey fills) parental geese during the three rearing phases. Scores were higher in males than in females, and decreased in males from phase 1 to phase 3

**Fig. 4:**
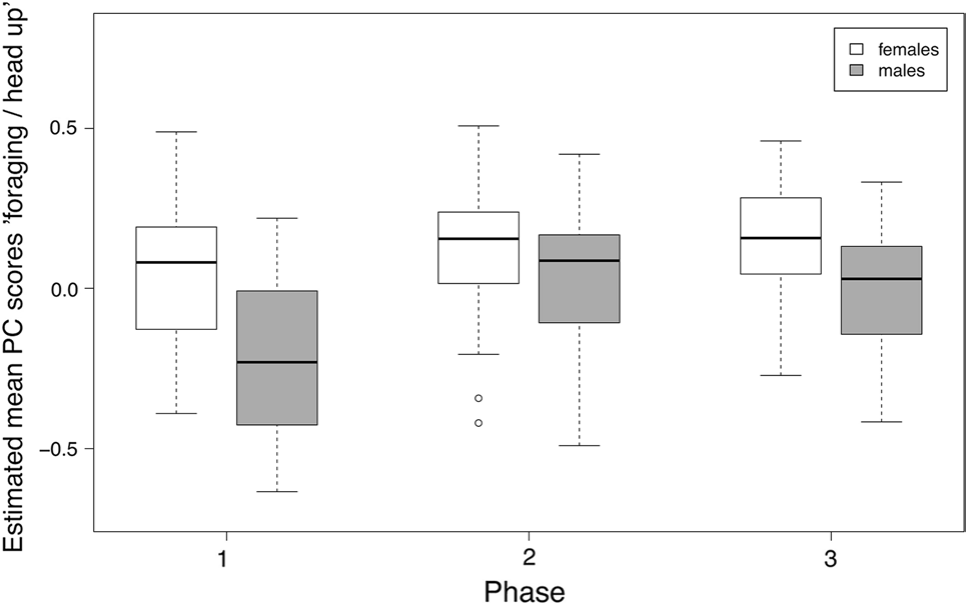
Estimated mean PC scores for foraging/head up in male (grey boxes) and female (white boxes) geese during the three rearing phases. An interaction between sex and phase was found, with a strong difference between males and females in phase 1

We found strong effects of sex, phase, and the number of goslings on general vigilance (Table 2). Scores of the general vigilance component were higher in males than in females. General vigilance increased in phase 2 and decreased again in phase 3 in males, while females stayed equally alert in phases 2 and 3, although to a lesser extent than males (Fig. 1). General vigilance also increased with increasing number of goslings (Fig. 2). The “agonistic interactions” component was strongly affected by sex, with higher scores in males than females. Scores in males decreased from phase 1 to phase 3 (Fig. 3). Furthermore, there was an effect of the number of goslings, with higher scores in agonistic interactions when the number of goslings was high (see Fig. 2). Finally, the foraging/head up component was influenced by the interaction between sex and phase, with a strong difference between males and females in phase 1 (Fig. 4). Females had a positive score while males had a negative score in phase 1, indicating that females were more often feeding whereas males showed more head up behaviour during the early phase of gosling rearing. Furthermore, there was an effect of the number of goslings, with decreasing scores as the number of goslings increased (Fig. 2). This finding indicates that head up, which loaded negatively on this component, increased, and foraging, which had a positive loading, decreased with higher numbers of goslings.

**Table 2:** Models with highest support and averaged models for the three principal components

	Estimate	SE	*t* value	CI (2.5%)	CI (97.5%)
*“General vigilance” component*					
(Intercept)	− 1.08	0.18	− 5.99	− 1.46	− 0.72
Phase (1 vs. 2)	0.63	0.13	4.89	0.37	0.88
Phase (1 vs. 3)	0.67	0.14	4.84	0.39	0.95
Sex (female vs. male)	0.81	0.17	4.72	0.46	1.16
Number of goslings	0.11	0.03	3.73	0.05	0.17
Phase 2: sex	0.06	0.17	0.35	− 0.27	0.39
Phase 3: sex	− 0.45	0.17	− 2.60	− 0.79	− 0.11

	Estimate	Adjusted SE	*z* value	CI (2.5%)	CI (97.5%)
*“Agonistic interactions” component*					
(Intercept)	− 0.43	0.18	2.43	− 0.78	− 0.08
Phase (1 vs. 2)	0.08	0.14	0.56	− 0.20	0.36
Phase (1 vs. 3)	0.05	0.15	0.34	− 0.25	0.36
Sex (female vs. male)	0.71	0.15	4.60	0.41	1.01
Number of goslings	0.06	0.03	1.97	0.00	0.11
Phase 2: sex	− 0.24	0.19	1.28	− 0.61	0.13
Phase 3: sex	− 0.51	0.19	2.67	− 0.89	− 0.14
*“Foraging/head up” component*					
(Intercept)	0.39	0.14	2.86	0.12	0.66
Phase (1 vs. 2)	− 0.08	0.15	0.52	− 0.36	0.21
Phase (1 vs. 3)	− 0.09	0.15	0.59	− 0.39	0.21
Sex (female vs. male)	− 0.28	0.15	1.82	− 0.58	0.02
Number of goslings	− 0.08	0.03	2.83	− 0.13	− 0.02
Phase 2: sex	0.44	0.19	2.27	0.06	0.82
Phase 3: sex	0.31	0.20	1.57	− 0.08	0.70

Used as reference: phase: phase 1, sex: females, “:” indicates interactions between factors

Estimated means, standard errors (SE), *t* values and confidence intervals (CI) are given. In averaged models, adjusted SE and *z* values are shown

**Fig. 5:**
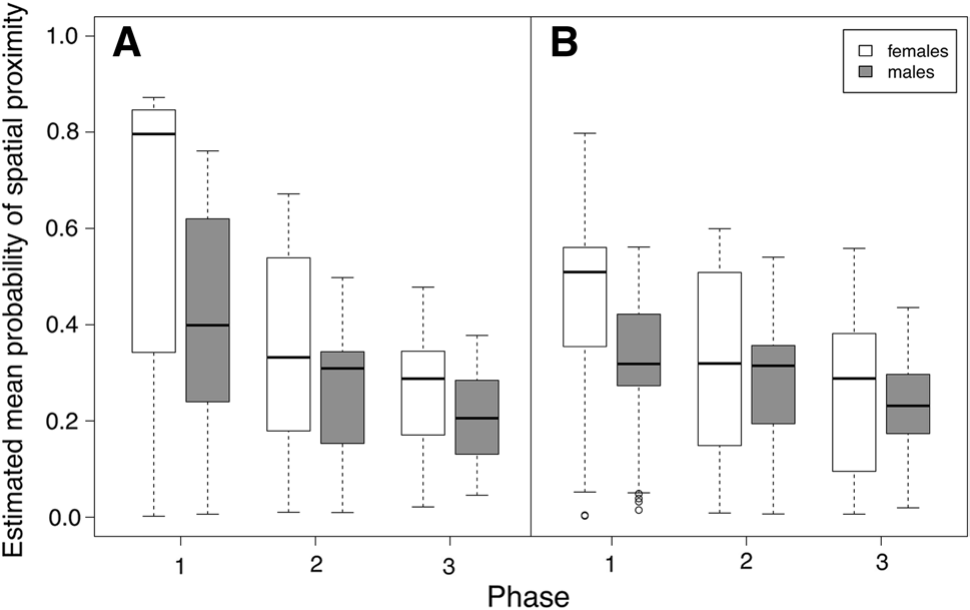
Estimated spatial proximity between female and male parental geese and their goslings during active (**a**) and inactive (**b**) phases during the three rearing phases. Spatial proximity decreased throughout the rearing phases, and goslings were closer to their mothers than to their fathers in phase 1

During both active and inactive phases, the rearing phase, sex, its two-way interaction, and the number of goslings had strong effects on the spatial proximity between parents and their goslings (Table 3). Goslings were more likely to stay closer to their parents in phase 1 than in phases 2 and 3, indicating that with age, goslings disperse further from their parents (Fig. 5). Sex also had a strong effect, and goslings were found in closer proximity to their mother as compared to their father during both, active and inactive phases. The interaction effect between rearing phase and sex showed that mothers were closer to their goslings especially during early rearing periods (phase 1), while spatial proximity to their goslings decreased in later rearing periods (see Fig. 5). The number of goslings influenced spatial proximity, and goslings were more likely to be in close proximity to their parents if the number of goslings was high.

**Table 3:** Models with highest support and averaged models for family proximity

	Estimate	Adjusted SE	*z* value	CI (2.5%)	CI (97.5%)
*Active phase*					
Phase (1 vs. 2)	0.81	0.17	4.63	0.47	1.15
Phase (1 vs. 3)	1.14	0.20	5.85	0.76	1.53
Sex (female vs. male)	0.64	0.17	3.64	0.29	0.98
Number of goslings	− 0.16	0.04	4.57	− 0.23	− 0.09
Phase 2: sex	− 0.35	0.20	1.73	− 0.74	0.05
Phase 3: sex	− 0.43	0.20	2.12	− 0.83	− 0.03

	Estimate	SE	*t* value	CI (2.5%)	CI (97.5%)
*Inactive phase*					
Phase (1 vs. 2)	1.15	0.15	7.44	0.84	1.45
Phase (1 vs. 3)	1.41	0.16	8.74	1.09	1.72
Sex (female vs. male)	1.50	0.17	8.60	1.16	1.84
Number of goslings	− 0.10	0.03	− 3.45	−0.15	− 0.04
Phase 2: sex	− 1.08	0.20	− 5.41	−1.47	− 0.69
Phase 3: sex	− 0.96	0.20	− 4.75	−1.36	− 0.57

Used as reference: phase: phase 1, sex: females, “:” indicates interactions between factors

Estimated means, standard errors (SE), *t* values and confidence intervals (CI) are given. For the averaged model, adjusted SE and *z* values are shown

**Fig. 6:**
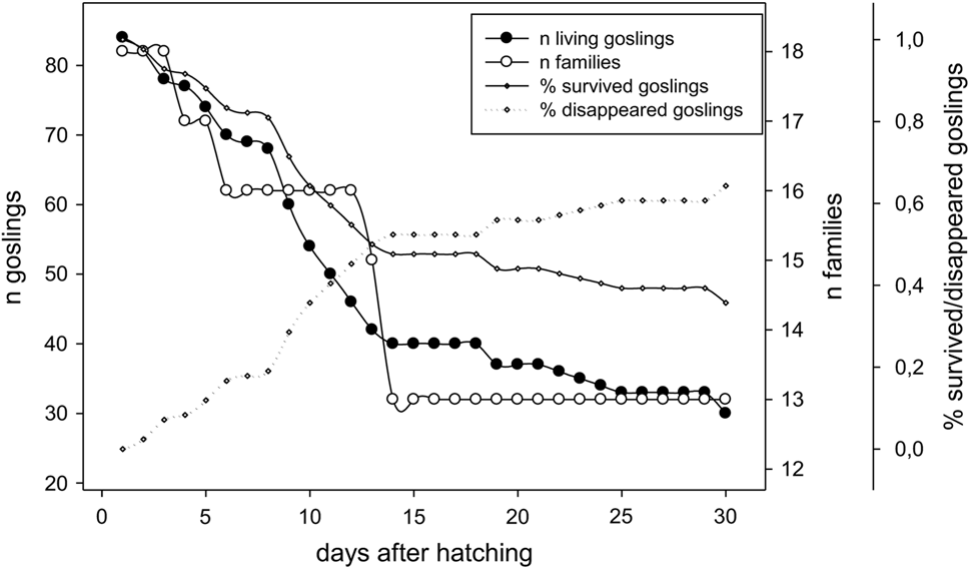
Development of the number of goslings (black circles) and families (white circles), and percentage of goslings that survived (diamond with full line) and disappeared (diamond with dotted line) during the rearing period (days after hatching)

Most goslings were lost within their first 10–15 days after hatching (Fig. 6). Gosling survival was strongly influenced by the agonistic interactions component and the foraging/head up component (Table 4). While gosling survival was more likely with increasing aggression, a negative relationship was found for survival and the foraging/head up components. This indicates that goslings had a higher chance of survival in more aggressive families and when the parents invested less in foraging and more in vigilance behaviour.

**Table 4:** Full and null model investigating gosling survival, with coefficients, estimated means, standard errors (SE), *t* values and confidence intervals (CI)

	Estimate	SE	*z* value	CI (2.5%)	CI (97.5%)
*Null model*					
(Intercept)	1.27	0.35	3.59	0.55	2.07
*Full model*					
(Intercept)	1.27	0.35	3.67	0.56	2.05
General vigilance component	− 0.02	0.07	− 0.25	− 0.15	0.12
Agonistic interactions component	0.14	0.07	2.00	0.00	0.28
Foraging/head up component	− 0.22	0.06	− 3.32	− 0.35	− 0.09

## Discussion

Our results indicate that parental investment, as indicated by quantitative behavioural differences within and between pairs, is critical for gosling survival. We found strong behavioural differences between male and female parental geese during the rearing of the young, with males more often being vigilant and aggressive towards nearby flock members (not their own family members), while females were more often foraging. These behavioural differences were most pronounced during the first 10 days after hatching (rearing phase 1). Furthermore, the more aggressive and vigilant the parents were, the more likely the goslings were to survive. This points to the importance of the basic trade-off between vigilance and feeding (Lima and Dill 1990): parents who allocate more time to vigilance raise a proportionally greater number of offspring than less vigilant parents.

In socially monogamous avian species, both parents participate in the majority of activities required for rearing the young (Trivers 1972). This is indeed more conspicuous in altricial than in precocial species, as in the former, parental care requires specific behaviours (i.e. food provisioning, e.g. Bart and Tornes 1989; Ligon 1999; Davies et al. 2012). In precocial birds, brood care activities are less obvious, since most of the parents’ behaviours are also performed in the absence of the offspring, apart from brooding, which is often a typical female behaviour (Lorenz 1988). For instance, experimental studies in precocial bird species found no difference in reproductive success between female Willow Ptarmigans (*Lagopus lagopus*) according to the presence of a male partner (Hannon 1984; Martin and Cooke 1987), and in the Lesser Snow Goose, females whose males were removed produced a comparable number of hatchlings as paired females (Martin et al. 1985). However, increased aggressive and vigilance behaviour in the presence of unfledged offspring is well known in ganders of several goose species as compared to males without offspring [Bar-headed Goose (Lamprecht 1986); White-fronted Goose, *Anser albifrons* (Boyd 1953; Stroud 1982); Canada Goose, *Branta canadensis* (Raveling 1970); Barnacle Goose, (Owen 1972; Black and Owen 1989)]. Here, we showed that vigilance and aggression not only increased with the number of goslings (e.g. Forslund 1993; Williams et al. 1994; Loonen et al. 1999), but actually seem to be the key to gosling survival. Sexes take different roles in this: ganders were aggressive and vigilant significantly more often than their females during the early rearing period. Therefore, we suggest that the males’ behaviour may be interpreted as “parental” and may, in fact, serve different functions, such as predator avoidance, defence against conspecifics, and allowing the offspring and the female to forage and rest undisturbed. This behaviour ensures that goslings survive the critical early rearing phase, and in addition, it allows the female to recover from the loss of body reserves suffered during incubation (Raveling 1979; Dittami 1981; Thompson and Raveling 1987).

Goslings were more likely to be found in close proximity to their mother than to their father in both inactive and active situations. This might be a consequence of the brooding activities exclusively performed by the female. On the other hand, the vulnerable goslings might learn to keep their distance from aggression, as males are more aggressive, and defend the family. The minimum distances between the female and their goslings confirm our expectation and the major role of the female in modulating the spatial distribution, and thus the social cohesion, of the family. In Greylag Geese, Scheiber and Weiß (2013) suggested that females were the driving force in structuring groups. Extended family bonds and spatial proximity between close female relatives indicate that females gain benefits through their maternal lineage. On the contrary, there is no evidence for sons or brothers seeking the proximity of their fathers/brothers in the same way adult females do. This is probably due to the benefits females gain through social support by relatives: reduced stress and increased feeding opportunities, and thus a better condition, higher fitness and higher reproductive success (Scheiber and Weiss 2018).

Note that when tested with distress calls of goslings, the females rather than the males responded with extreme head up (Loth et al. 2017). This further emphasizes the different roles males and females assume during gosling rearing: while females invest more in guarding and leading the offspring, males invest more in guarding the female, pay more attention to the social environment, and provide the female with feeding opportunities. How these behavioural differences are modulated by the endocrine system (e.g. steroid hormones) is well-documented (Nelson 1995; Hirschenhauser et al. 2013). In Greylag Geese, results from long-term research showed significant differences in both hormonal patterns and heart rates within the sexes depending on breeding and reproductive success (Kotrschal et al. 1998, 2000; Hirschenhauser et al. 1999a, b; Hirschenhauser et al. 2000; Frigerio et al. 2004; Scheiber et al. 2005; Wascher et al. 2008, 2012). In addition to hormonal patterns, effects of age and bonding duration may influence the behavioural coordination (Fowler 1995). For example, geese with longer pair bonds were found to produce more offspring in Barnacle Geese (Black 2001) and have higher clutch sizes in Lesser Snow Geese (Cooke et al. 1981), while there is no evidence in other socially monogamous species (Griggio and Hoi 2011). Evidence in Greylag Geese is still missing, and we could not account for these effects in this study without critically reducing the sample size, but geese that are older or paired longer may be better attuned to each other, and thus more successful in raising goslings.

## Conclusions

Our study provides insight into the different roles of male and female pair partners during rearing of the young in the socially complex Greylag Goose, and how behavioural investment, particularly in agonistic and vigilance behaviour, affects gosling survival. During the early stages of rearing, when young are particularly vulnerable and inexperienced, males are highly vigilant, thereby evidently increasing the goslings’ odds of surviving the critical first weeks. Males are also aggressive towards other flock members in this stage of rearing, thus preventing other families from moving into close proximity, which might result in adoptions of goslings. Females, on the other hand, emphasize spatial cohesion with their goslings. We suggest that these specific sex roles represent a cooperation between pair partners towards optimizing offspring survival.

## Electronic supplementary material

Below is the link to the electronic supplementary material.

Supplementary material 1 (DOCX 17 kb)

## Data Availability

The data sets generated during and/or analysed during the current study are available from the corresponding author on reasonable request.
