# Supplementary material for: Parental behaviour and family proximity as key to gosling survival in Greylag Geese (Anser anser)
Source: J Ornithol. 2019 Feb 20;160(2):473–83. doi: 10.1007/s10336-019-01638-x (PMC6476843; doi:10.1007/s10336-019-01638-x)
Supplement: Supplementary file 1 — Supplementary material 1 (DOCX 17 kb) [file 10336_2019_1638_MOESM1_ESM.docx]

# Supplemental Materials

Table S1: Model selection table for the three Principal Components, showing AICc values for each model, the difference in AICc (ΔAICc), relative likelihood, and Akaike weights. Models with high support are indicated in bold.

| Model | AICc | ΔAICc | relative likelihood | Akaike weight |
| --- | --- | --- | --- | --- |
| *“general vigilance” component* |  |  |  |  |
| **phase*sex + number of goslings** | **1401.38** | **0.0** | **1.00** | **0.94** |
| phase + sex + number of goslings | 1406.93 | 5.55 | 0.06 | 0.06 |
| phase*sex | 1412.84 | 11.46 | 0.0 | 0.0 |
| phase + sex | 1418.12 | 16.74 | 0.0 | 0.0 |
| phase + number of goslings | 1420.89 | 19.51 | 0.0 | 0.0 |
| sex + number of goslings | 1445.43 | 44.06 | 0.0 | 0.0 |
| phase | 1433.02 | 31.64 | 0.0 | 0.0 |
| sex | 1443.76 | 42.38 | 0.0 | 0.0 |
| number of goslings | 1460.86 | 59.49 | 0.0 | 0.0 |
| Null model | 1459.00 | 57.62 | 0.0 | 0.0 |
| *“agonistic interactions” component* |  |  |  |  |
| **phase*sex + number of goslings** | **1507.97** | **0.0** | **1.00** | **0.40** |
| phase + sex + number of goslings | 1510.93 | 2.96 | 0.23 | 0.09 |
| **phase*sex** | **1508.80** | **0.83** | **0.66** | **0.26** |
| phase + sex | 1511.78 | 3.80 | 0.15 | 0.06 |
| phase + number of goslings | 1526.40 | 18.43 | 0.0 | 0.0 |
| **sex + number of goslings** | **1509.52** | **1.54** | **0.46** | **0.18** |
| phase | 1526.90 | 18.93 | 0.0 | 0.0 |
| sex | 1515.14 | 7.16 | 0.03 | 0.01 |
| number of goslings | 1525.00 | 17.02 | 0.0 | 0.0 |
| Null model | 1530.29 | 22.32 | 0.0 | 0.0 |
| *“foraging/head up” component* |  |  |  |  |
| **phase*sex + number of goslings** | **1547.32** | **0.85** | **0.65** | **0.24** |
| phase + sex + number of goslings | 1548.77 | 2.30 | 0.32 | 0.12 |
| phase*sex | 1550.50 | 4.02 | 0.13 | 0.05 |
| phase + sex | 1551.95 | 5.47 | 0.06 | 0.02 |
| phase + number of goslings | 1550.78 | 4.30 | 0.12 | 0.04 |
| **sex + number of goslings** | **1546.48** | **0.0** | **1.00** | **0.37** |
| phase | 1553.96 | 7.48 | 0.02 | 0.01 |
| sex | 1555.49 | 9.02 | 0.01 | 0.0 |
| number of goslings | 1548.50 | 2.02 | 0.36 | 0.14 |
| Null model | 1557.52 | 11.05 | 0.0 | 0.0 |

Table S2: Model selection table for the spatial proximity of parents to their goslings during active and passive phases. AICc values, the difference in AICc (ΔAICc), relative likelihood, and Akaike weights are shown for each model. Models with high support are indicated in bold.

| Model | AICc | ΔAICc | relative likelihood | Akaike weight |
| --- | --- | --- | --- | --- |
| *Active phase* |  |  |  |  |
| **phase*sex + number of goslings** | **2024.87** | **0.00** | **1.00** | **0.62** |
| **phase + sex + number of goslings** | **2025.86** | **0.98** | **0.61** | **0.38** |
| phase*sex | 2042.44 | 17.57 | 0.0 | 0.0 |
| phase + sex | 2043.51 | 18.63 | 0.0 | 0.0 |
| sex + number of goslings | 2082.48 | 57.60 | 0.0 | 0.0 |
| phase + number of goslings | 2041.01 | 16.13 | 0.0 | 0.0 |
| sex | 2195.65 | 170.78 | 0.0 | 0.0 |
| phase | 2058.53 | 33.65 | 0.0 | 0.0 |
| number of goslings | 2097.83 | 72.95 | 0.0 | 0.0 |
| Null model | 2211.29 | 186.41 | 0.0 | 0.0 |
| *Passive phase* |  |  |  |  |
| **phase*sex + number of goslings** | **2143.55** | **0.00** | **1.00** | **0.99** |
| phase + sex + number of goslings | 2175.21 | 31.66 | 0.0 | 0.0 |
| phase*sex | 2152.05 | 8.50 | 0.01 | 0.01 |
| phase + sex | 2183.70 | 40.15 | 0.0 | 0.0 |
| sex + number of goslings | 2232.71 | 89.16 | 0.0 | 0.0 |
| phase + number of goslings | 2203.24 | 59.69 | 0.0 | 0.0 |
| sex | 2292.41 | 148.86 | 0.0 | 0.0 |
| phase | 2207.70 | 64.15 | 0.0 | 0.0 |
| number of goslings | 2258.32 | 114.77 | 0.0 | 0.0 |
| Null model | 2317.11 | 173.56 | 0.0 | 0.0 |

## Behavioural observations

During phase 1, a minimum of 6 and a maximum of 8 scans were conducted per family (N_adults_ = 36, N_goslings_ = 84; average number of scans per family ± SE = 6.22 ± 0.13). During phase 2, a minimum of 1 and a maximum of 6 scans were done (N_adults_ = 32, N_goslings_ = 47; average number of scans per family ± SE = 5.31 ± 0.37). During phase 3, a minimum of 6 and a maximum of 7 scans were conducted (N_adults_ = 26, N_goslings_ = 33; average number of scans per family ± SE = 6.08 ± 0.07).

## Proximity data

During phase 1, a minimum of 7 and a maximum of 11 distance measures were collected per family during inactive periods (N_adults_ = 36, average number of measures per family ± SE = 8.33 ± 0.22) and a minimum of 8 and a maximum of 13 measures were collected per family during active periods (average number of measures per family ± SE = 8.89 ± 0.32). During phase 2, a minimum of 3 and a maximum of 10 measures were collected per family during inactive periods (N_adults_ = 32, average number of measures per family ± SE = 7.31 ± 0.51) and a minimum of 1 and a maximum of 9 measures were collected per family during active periods (average number of measures per family ± SE = 7.25 ± 0.52). During phase 3, a minimum of 8 and a maximum of 9 measures were collected per family during inactive periods (N_adults_ = 26, average number of measures per family ± SE = 8.31 ± 0.13) and a minimum of 8 and a maximum of 9 measures were collected per family during active periods (average number of measures per family ± SE = 8.08 ± 0.07).
